# Mechanical Properties and Corrosion Behavior of Biodegradable Metals: Current Challenges

**DOI:** 10.3390/ma19173646

**Published:** 2026-08-27

**Authors:** Fatemeh Nazeran, Seyed Mahmood Fatemi, Nafiseh Mollaei, Mohammad H. Pishbin, Jessica Calvo, Jose Maria Cabrera

**Affiliations:** 1 School of Metallurgy and Materials Engineering, College of Engineering, University of Tehran, Tehran P.O. Box 11155-4563, Iran; 2 Department of Materials Science and Engineering, Campus Diagonal Besòs-EEBE, Universitat Politècnica de Catalunya-BarcelonaTech, 08019 Barcelona, Spainjose.maria.cabrera@upc.edu (J.M.C.); 3 IMDEA Materials Institute, 28906 Getafe, Madrid, Spain; 4 Department of Materials Science, Universidad Politécnica de Madrid, 28040 Madrid, Spain; 5 Fundació Centre CIM, Llorens i Artigas 12, 08028 Barcelona, Spain

**Keywords:** biodegradable metals, corrosion behavior, mechanical properties, magnesium alloys, zinc alloys, iron alloys, biomaterials

## Abstract

Biodegradable metals have emerged as promising alternatives to permanent implants because they provide temporary mechanical support while gradually degrading after tissue healing, eliminating the need for removal surgery. Among the main biodegradable systems, Fe-, Mg-, and Zn-based alloys each offer unique advantages but also face important challenges related to the balance between mechanical performance, corrosion behavior, and biocompatibility. This review summarizes recent advances in the development of these materials, focusing on how alloy design, thermomechanical processing, porous architectures, surface engineering, and additive manufacturing influence their mechanical properties and degradation mechanisms. Special attention is given to the interplay between corrosion and mechanical integrity throughout the implant lifetime, as well as to current strategies for tailoring degradation rates to match tissue healing. Finally, the review discusses the remaining challenges, including the need for standardized testing, improved long-term in vivo validation, and the development of next-generation biodegradable metallic implants with optimized structural, biological, and functional performance.

## 1. Introduction

The aging global population has increased the demand for biodegradable metallic materials for temporary biomedical devices, particularly orthopedic implants and cardiovascular stents [1,2]. Unlike conventional permanent implants such as stainless steels, titanium alloys, and Co-Cr alloys, biodegradable metals provide temporary mechanical support and gradually degrade after tissue healing, eliminating the need for secondary removal surgeries and reducing long-term complications such as stress shielding, chronic inflammation, implant failure, and impaired tissue remodeling [1,3]. Biodegradable metals are mainly classified into Fe-, Mg-, and Zn-based systems, each exhibiting distinct advantages and limitations. Fe-based alloys offer excellent mechanical strength but degrade too slowly in physiological environments, potentially leading to long-term persistence of corrosion products [3,4]. Mg-based alloys possess an elastic modulus closer to bone, reducing stress shielding, but their highly negative electrochemical potential causes excessively rapid degradation and hydrogen evolution, which may compromise mechanical integrity and induce gas-related complications [1,5,6,7]. Zn-based alloys have emerged as an attractive intermediate solution, exhibiting degradation rates between those of Fe and Mg while avoiding hydrogen generation. Zinc also plays important physiological roles in tissue regeneration and cellular activity. However, pure Zn generally exhibits insufficient strength and limited ductility in the as-cast state, restricting its direct use in load-bearing applications. Nevertheless, its ductility and mechanical performance are strongly processing-dependent and can be significantly improved by extrusion, rolling, drawing, and other thermomechanical routes [8,9,10].

**Figure 1:**
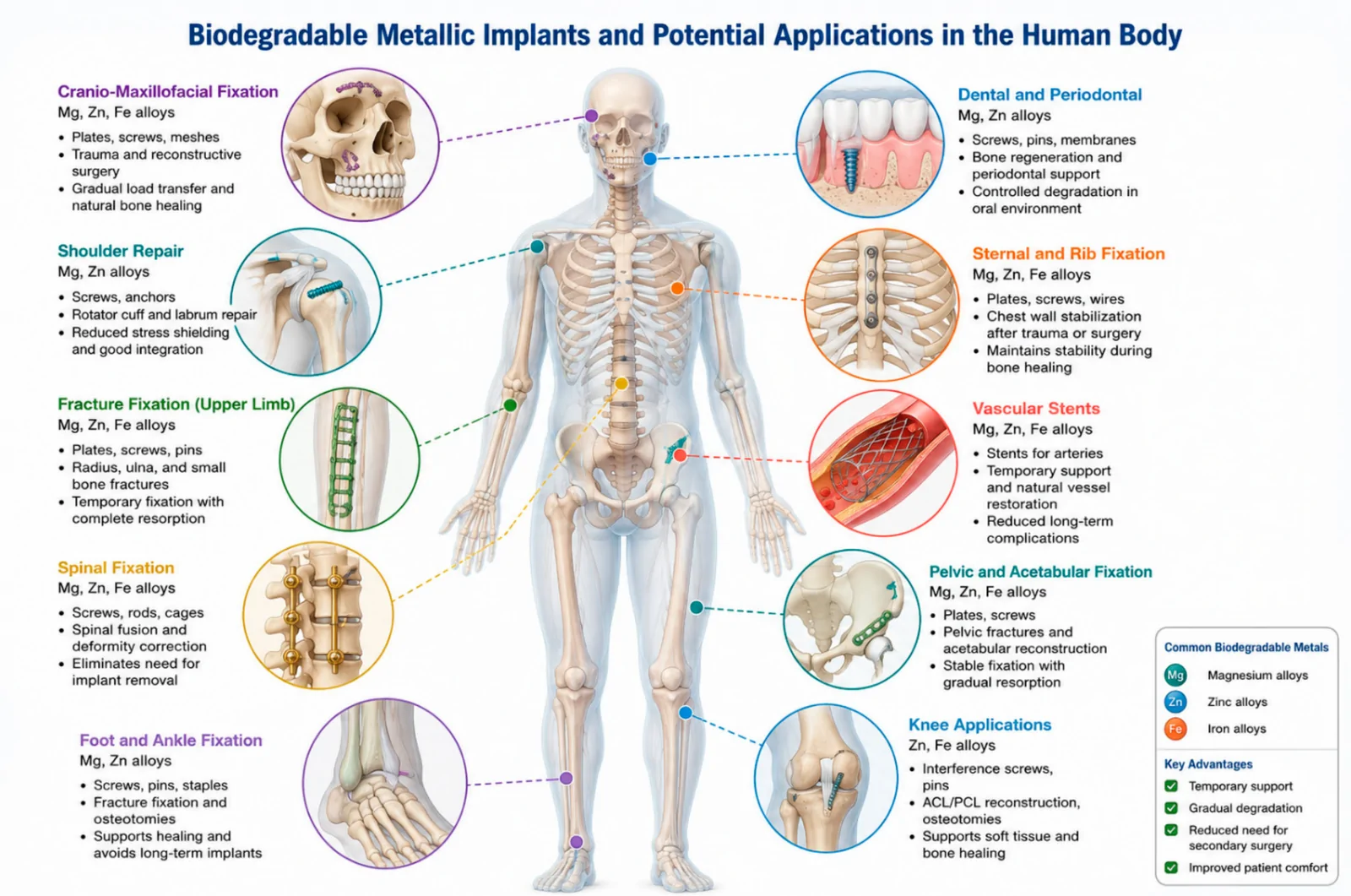
Schematic illustration of the potential biomedical applications of biodegradable metallic implants in the human body. Based on Refs. [29,30].

A major challenge in biodegradable metallic systems is achieving a balance between mechanical integrity, corrosion behavior, and biocompatibility, as these factors are strongly interconnected. During degradation, microstructural changes, pore formation, cracking, and corrosion products progressively reduce mechanical performance, making it essential to maintain structural integrity throughout the healing period [3,11]. To address these limitations, significant efforts have focused on alloy design, thermomechanical processing, microstructural engineering, surface modification, and additive manufacturing. These approaches aim to tailor degradation behavior, improve mechanical properties, and enable the fabrication of patient-specific implants with controlled architectures and porosity [1,2,11]. Bioresorbable metallic alloys are commonly produced using casting, powder metallurgy, thermomechanical processing, surface modification, and additive manufacturing routes [1,2,3,11]. Casting is widely used for initial alloy development but often produces coarse grains, segregation, and brittle secondary phases [1,3,11]. Thermomechanical processing, including rolling, extrusion, forging, drawing, ECAP, and HPT, is applied to refine grains, redistribute intermetallic phases, modify texture, and improve the strength–ductility–corrosion balance [12,13,14,15]. Powder metallurgy and space-holder methods enable porous architectures with controlled pore size and porosity [16,17,18,19], while additive manufacturing techniques such as LPBF, SLM, binder jetting, and extrusion-based 3D printing provide greater control over patient-specific geometry and scaffold topology [20,21,22,23,24]. Surface treatments, including plasma electrolytic oxidation, hydroxyapatite-based coatings, and other protective conversion layers, are frequently combined with bulk processing to regulate early-stage degradation and biological response [25,26,27]. More recently, hybrid and multi-material systems combining different biodegradable metals or metal-polymer combinations have been proposed to achieve optimized mechanical performance, degradation kinetics, and biological functionality [3]. Despite recent progress, the relationships among processing methods, microstructure evolution, corrosion behavior, mechanical degradation, and biological performance are still not fully understood. In addition, rapid advances in alloy development, thermomechanical processing, porous structures, and additive manufacturing have generated a large and scattered body of knowledge across different biodegradable metallic systems. Therefore, a comprehensive assessment of current progress and remaining challenges is still needed. Unlike previous reviews, which have primarily examined alloy composition, degradation behavior, or additive manufacturing as separate topics [1,2,3,20,21], this article focuses on the interrelationship among processing routes, microstructural evolution, corrosion kinetics, and the preservation of mechanical integrity in iron-, magnesium-, and zinc-based biodegradable metals. Attention is also given to thermomechanical processing regimes, heat treatments, porous architectures, and additive manufacturing strategies to clarify how processing-induced grain refinement, crystallographic texture, secondary phases, and scaffold topology can simultaneously govern degradation behavior and load-bearing performance [12,13,14,15,28]. By integrating processing–structure–property relationships, this review provides a more analytical perspective for the design of the next generation of biodegradable metallic implants. This review focuses on the corrosion behavior and mechanical performance of Fe-, Mg-, and Zn-based biodegradable metals, highlighting the effects of alloying, processing routes, porous architectures, and additive manufacturing on their overall performance. Finally, it discusses the main challenges and future opportunities for developing next-generation biodegradable metallic biomaterials [2,3,11]. The potential biomedical applications of biodegradable metallic implants in different regions of the human body are schematically illustrated in Figure 1.

## 2. Standard Requirements and Design Criteria for Biodegradable Metals

Unlike conventional biomaterials such as titanium alloys and stainless steels, biodegradable metals must be evaluated not only for their initial mechanical properties and biocompatibility but also for the changes that occur during in vivo degradation [2,3,11]. These changes include degradation kinetics, corrosion product formation, ion release, hydrogen evolution, and the gradual loss of mechanical integrity. To address these challenges, standards, including ISO/TS 20721 and ASTM F3268, were specifically established for absorbable metallic implants. These standards consider key aspects such as degradation kinetics, corrosion–mechanical interactions, ion release, hydrogen evolution, and time-dependent property changes. Their goal is to assess the safety and functionality of biodegradable metals under physiologically relevant conditions [31,32].

However, many studies continue to employ conventional standards originally developed for permanent implants or engineering materials, such as ASTM E8 [33] for tensile testing, ASTM G31 [34] for immersion corrosion testing, and ISO 10993 [35] for biological evaluation. While these methods provide reliable and reproducible and well-established procedures, they do not fully represent the dynamic degradation processes of biodegradable metals. For example, tensile tests measure only the initial mechanical properties, whereas conventional corrosion tests often fail to reproduce the complexity of the physiological environment or the simultaneous interaction between corrosion and mechanical degradation [32,33,34,35].

As a result, current assessment of biodegradable metals generally relies on a combination of dedicated biodegradable-metal standards and adapted conventional methodologies. This hybrid approach remains necessary because no single standard currently addresses all aspects of biodegradable implant performance. Future standardization efforts should focus on integrating mechanical, corrosion, and biological evaluations to enable more reliable prediction of long-term clinical performance [3,31,32,35].

### 2.1. Mechanical Performance Criteria

The mechanical requirements of biodegradable metallic implants depend strongly on their intended application. In orthopedic devices, materials must provide sufficient load-bearing support during bone healing while minimizing stress shielding through an elastic modulus closer to that of cortical bone [1,2]. For vascular stents, materials must combine adequate radial strength, high ductility for expansion, low elastic recoil, and fatigue resistance under cyclic loading [4,9].

For biodegradable stents, typical benchmark requirements include yield strength exceeding ~200 MPa, ultimate tensile strength above ~300 MPa, and elongation to failure greater than 15–20% to withstand crimping and balloon expansion without fracture. Elastic recoil should generally remain below 4% to ensure effective vessel support. Young’s modulus must also be optimized to balance radial strength and mechanical compatibility with the surrounding tissues [4,9,36].

**Figure 2:**
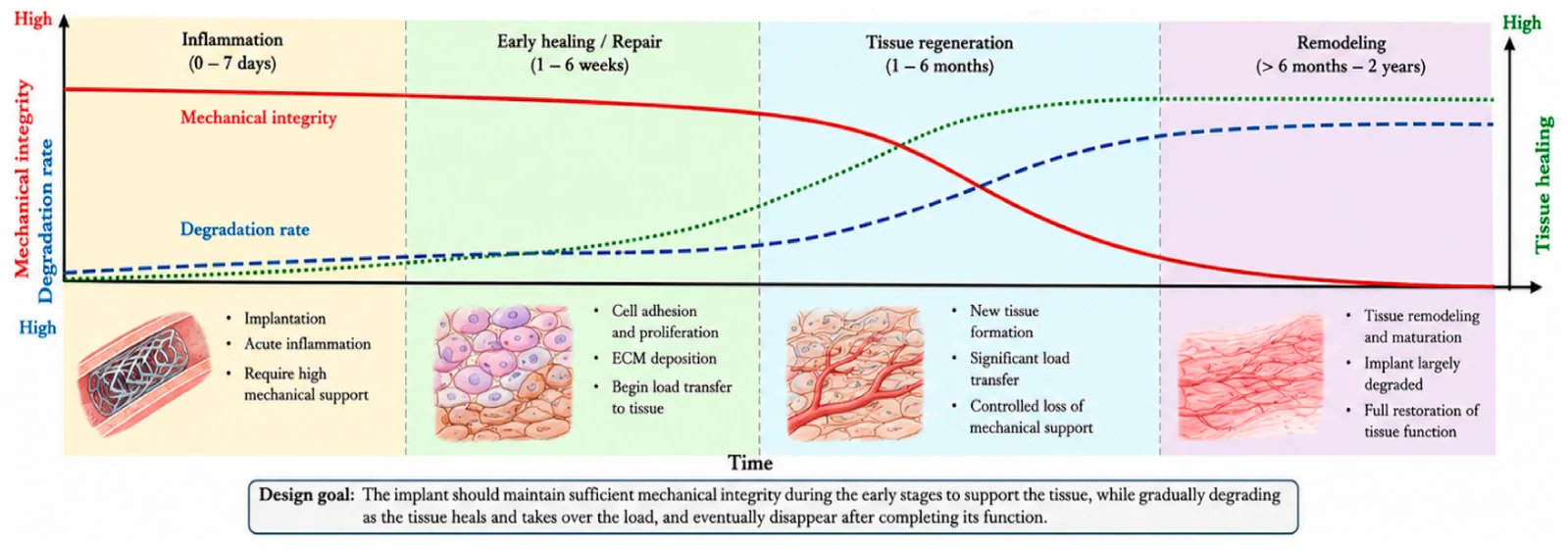
Schematic illustration of the relationship between implant degradation, mechanical integrity, and tissue healing during the implantation period. Based on Refs. [37,38].

In orthopaedic applications, implants must maintain mechanical integrity throughout healing while progressively transferring load to the regenerating tissue. Therefore, degradation behavior and mechanical integrity should evolve in a synchronized manner, as illustrated in Figure 2. Premature degradation may result in the loss of mechanical support and implant failure, whereas excessively slow degradation reduces the clinical benefits of biodegradable systems [1,3,11]. Table 1 summarizes the mechanical benchmark criteria for biodegradable orthopedic implants and vascular stents.

Table 2 compares the typical mechanical properties of Mg-, Zn-, and Fe-based biodegradable metals with cortical bone, highlighting the challenge of simultaneously achieving suitable strength, ductility, stiffness, and degradation behavior. Fe-based alloys provide the highest strength but are limited by their high elastic modulus and slow degradation rate. Mg alloys exhibit a bone-like modulus but often degrade too rapidly, leading to premature loss of mechanical support. Zn alloys offer intermediate degradation behavior and good biocompatibility without hydrogen evolution; however, pure Zn still shows limited strength and ductility, motivating extensive research on alloying strategies and thermomechanical processing routes to enhance its mechanical performance while maintaining favorable biodegradation characteristics [1,8,9,10]. Overall, these benchmark values should be regarded as application-dependent guidelines rather than fixed acceptance limits. Several Fe-, Mg-, and Zn-based biodegradable alloys have approached the required mechanical ranges after appropriate alloying and processing; however, meeting the initial strength requirement alone is insufficient. For biodegradable implants, mechanical performance must be evaluated together with degradation rate, corrosion mode, retention of mechanical integrity, and biological response over time. Therefore, materials that satisfy tensile or compressive benchmarks in the undegraded state may still be unsuitable if rapid localized corrosion, excessive gas evolution, or long-term accumulation of corrosion products compromises their in vivo functionality.

Although Table 2 summarizes the baseline mechanical properties of pure Fe, Mg, and Zn, alloying and processing can substantially modify these properties and bring selected systems closer to application-specific mechanical benchmarks. Therefore, representative Fe-, Mg-, and Zn-based biodegradable systems that approach these requirements after alloying and processing, together with their main advantages and remaining limitations, are summarized in Table 3.

This comparison indicates that meeting the initial mechanical benchmark is necessary but not sufficient, because each alloy family still faces specific degradation-related limitations that must be considered together with mechanical performance.

### 2.2. Corrosion Requirements: Pure Fe, Mg, and Zn

The corrosion behavior of biodegradable metals plays a critical role in determining both their mechanical integrity and biological performance. Unlike permanent implant materials, biodegradable metals are designed to undergo controlled degradation in physiological environments, progressively transferring load to the healing tissue before complete resorption. Consequently, the degradation rate must be carefully tailored to the tissue healing process to prevent either premature loss of mechanical support or excessive implant retention [3,11].

**Figure 3:**
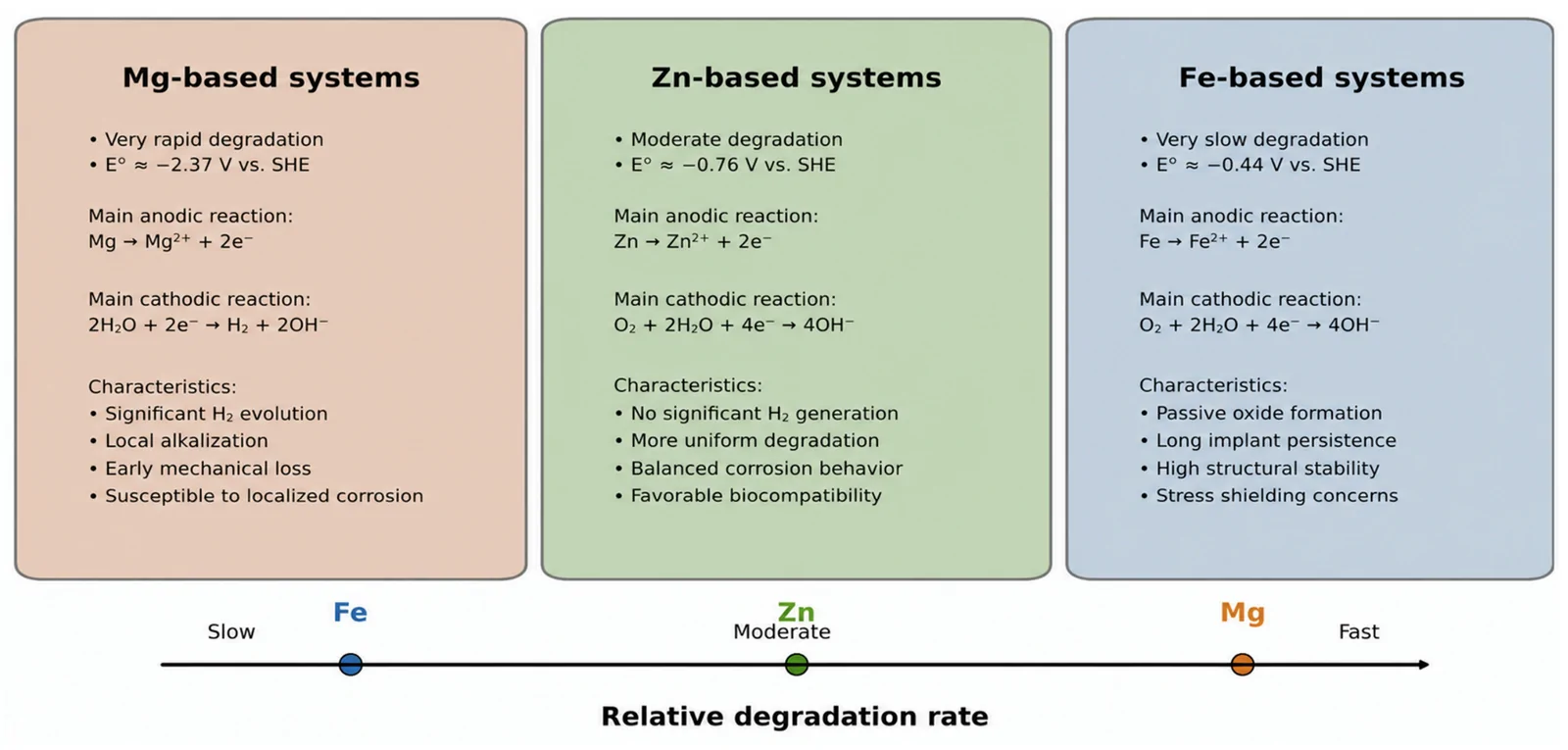
Comparison of the degradation behavior of Fe, Mg, and Zn-based biodegradable metals in physiological environments, including electrochemical reactions, relative degradation rates, and mechanical integrity retention during implantation. Detailed corrosion products are summarized in Table 4. Based on Refs. [38,43].

A comparison of the electrochemical and corrosion characteristics of Fe-, Mg-, and Zn-based biodegradable metals is provided in Table 4, while Figure 3 schematically summarizes the corresponding degradation mechanisms, relative degradation rates, hydrogen-gas evolution, and mechanical-integrity retention. As shown in Table 4 and Figure 3, Fe-based systems generally corrode slowly because of passive oxide/hydroxide film formation, Mg-based systems exhibit rapid dissolution accompanied by hydrogen evolution and early mechanical loss, and Zn-based systems show an intermediate degradation tendency without significant gas generation. However, Zn corrosion should not be considered intrinsically uniform, because alloy composition, secondary-phase distribution, crystallographic texture, processing route, and local galvanic coupling can produce heterogeneous or localized corrosion. Therefore, Zn degradation is better described as moderate but microstructure-dependent rather than uniformly homogeneous [1,5,6,42,43].

To address these limitations, considerable research has focused on alloy design, microstructural engineering, surface modification, and thermomechanical processing. More recently, additive manufacturing and functionally graded biodegradable materials have attracted increasing interest, enabling patient-specific degradation profiles through spatial control of porosity, microstructure, and corrosion behavior within a single implant [2,11].

The measured corrosion behavior in biodegradable Mg-, Zn-, and Fe-based metals strongly depends on the selected physiological test medium (Table 5). Commonly used solutions such as SBF, HBSS, PBS, DMEM, Ringer’s solution, and Tyrode’s solution differ in buffering capacity, chloride concentration, bicarbonate content, phosphate level, calcium ions, proteins, amino acids, and organic components. These differences can directly affect pH evolution, corrosion-product formation and stability, surface passivation, localized corrosion tendency, and the apparent degradation rate [44,45,46,47].

Therefore, corrosion rates reported for biodegradable metals should not be compared without considering the test medium, pH control, solution renewal, immersion time, temperature, surface-area/solution-volume ratio, and the presence or absence of organic components. This issue is particularly important for Mg alloys because hydrogen evolution and alkalization are highly sensitive to buffering capacity, but it is also relevant for Zn and Fe alloys because phosphate, carbonate, chloride, and protein-containing species can alter corrosion-product stability and surface passivation [8,12,44,45,46,47].

### 2.3. Biocompatibility

Biocompatibility is a fundamental requirement for biodegradable metals because both the implant and its degradation products interact continuously with surrounding tissues. Unlike permanent implants, biodegradable materials release ions and corrosion products during degradation, making their biological response dynamic and time-dependent [3,11,35].

Magnesium and zinc offer clear advantages as they are essential elements involved in bone metabolism, enzymatic activity, cell proliferation, immune response, and tissue regeneration. Consequently, their degradation products are generally well-tolerated and may even promote healing when released in controlled amounts. Iron is also an essential element; however, its slow degradation may lead to the accumulation of corrosion products, potentially affecting tissue remodeling and causing localized inflammatory responses [5,48,49].

Recent developments increasingly focus on biofunctionalization, where biodegradable metals are designed not only to be biocompatible but also to actively stimulate biological processes. This includes alloying strategies to enhance antibacterial activity, promote cell growth, and improve tissue integration, as well as surface treatments that regulate ion release and biological interactions. Another emerging trend is the integration of mechanical and biological design, where degradation is tailored to release beneficial ions at specific stages of healing. These developments reflect the transition from passive biomaterials toward multifunctional implants. The strong interrelationship between mechanical properties, corrosion behavior, and biocompatibility highlights the need for a holistic design approach, combining alloy development, processing, and surface engineering to achieve optimal performance [1,2,3,11].

## 3. Fe-Based Alloys

### 3.1. Overview and Main Limitations

Fe based biodegradable alloys have attracted considerable attention owing to their excellent mechanical properties, including high strength, good ductility, and superior fatigue resistance. These characteristics make them attractive candidates for load-bearing orthopedic implants and cardiovascular stents. In addition, iron is an essential trace element involved in numerous physiological processes, providing an inherent advantage from a biological perspective [12,40,42,50].

Despite these benefits, the clinical application of Fe-based biodegradable implants remains limited by their extremely slow degradation rate in physiological environments. The slow corrosion behavior of Fe is primarily associated with the formation of stable passive oxide layers that protect the surface from further degradation. While such passivation is beneficial for permanent implants, it is undesirable for biodegradable systems, where controlled resorption is required. As a result, Fe implants may persist for several years after implantation, exceeding the intended healing period [49,51,52]. Furthermore, the high elastic modulus of Fe (~200 GPa) is substantially higher than that of cortical bone (10–30 GPa), increasing the risk of stress shielding and subsequent bone resorption. The accumulation of iron oxides and hydroxides may also interfere with tissue remodeling and induce localized biological responses. Consequently, extensive research has focused on accelerating the degradation of Fe-based materials while preserving their favorable mechanical properties and biocompatibility [42,49,50]. From a clinical perspective, the main difficulty in Fe-based biodegradable alloys is not the lack of initial mechanical strength, but the mismatch between their slow degradation kinetics and the required healing time of temporary implants. Although alloying and processing can improve strength, ductility, and corrosion activity, Fe-based systems often remain protected by relatively stable oxide/hydroxide layers, which limit ion release and delay complete resorption. This slow degradation may reduce the biological advantage of a bioresorbable implant and increase the risk of long-term corrosion-product retention, stress shielding, and incomplete tissue remodeling. Therefore, future Fe-based implant design should not focus only on increasing corrosion rate, but should also consider corrosion uniformity, corrosion-product transport, local tissue response, magnetic compatibility, and the retention of mechanical integrity during the intended healing period [4,12,40].

### 3.2. Development: Fe Alloying, Bulk Processing, and Porous Architectures

The development of Fe-based biodegradable alloys has followed two complementary strategies: The first strategy focuses on dense bulk components, in which alloying and thermomechanical processing are used to modify phase stability, refine the microstructure, improve mechanical properties, and accelerated degradation. The second strategy involves the design of porous Fe-based scaffolds, where controlled porosity and interconnected pore networks increase the exposed surface area, facilitate electrolyte penetration, reduce stiffness and promote tissue ingrowth. Together, these approaches address the intrinsic limitation of Fe systems, namely their excessively slow degradation compared with clinical healing times [12,20,50,53].

**Figure 4:**
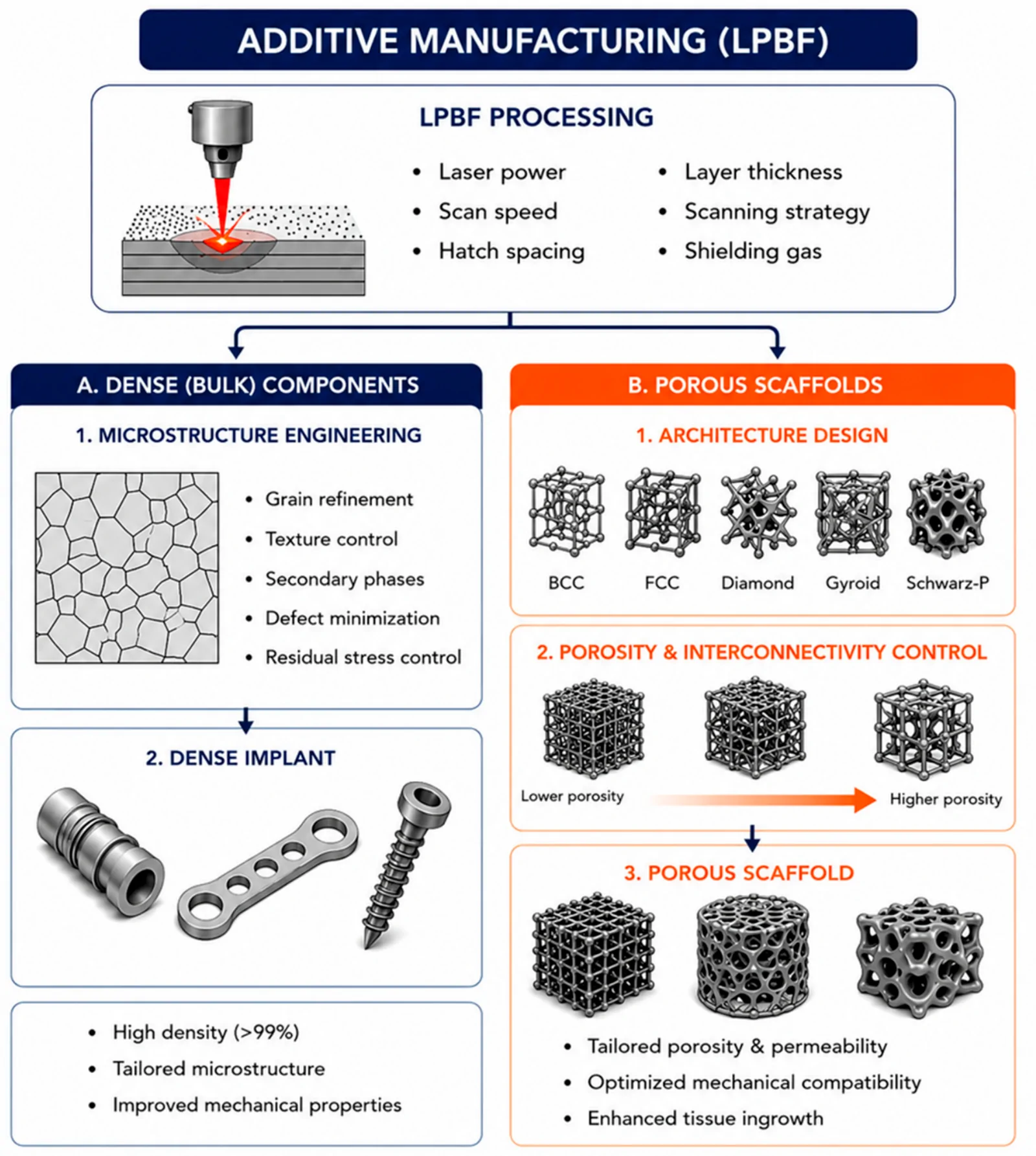
Schematic illustration of the main development routes for additively manufactured biodegradable metallic implants. Dense components are optimized through alloy design and microstructure control, while porous scaffolds are designed by tailoring architecture, porosity, and interconnectivity. Based on Refs. [54,55,56].

As schematically illustrated in Figure 4, biodegradable metallic systems can generally be developed through two complementary routes: dense bulk components produced via microstructural engineering and porous scaffolds designed through architectural control.

#### 3.2.1. Alloying Strategies in Fe-Based Bioresorbable Alloys

Alloying remains one of the most effective approaches for improving the performance of biodegradable Fe-based alloys. Because the primary limitation of pure Fe is its extremely slow degradation rate, alloy design has largely focused on accelerating corrosion while preserving the excellent mechanical properties that make Fe attractive for biomedical applications. Depending on their primary function, alloying elements can generally be categorized as austenite stabilizers, degradation accelerators, and biofunctional elements [40,52,57].

Mn, C, and Si are among the most widely investigated alloying additions in Fe-based bioresorbable alloys because they simultaneously influence phase stability, mechanical behavior, magnetic response, and corrosion activity. Mn is particularly important because it stabilizes the γ-austenite phase, reduces ferromagnetism, and improves ductility, making Fe–Mn alloys one of the benchmark systems for biodegradable implant applications [40,51,58]. Carbon further enhances strength and work hardening in Fe–Mn–C alloys through solid-solution strengthening and TWIP/TRIP-assisted deformation mechanisms; however, carbide formation must be carefully controlled because it may introduce local micro-galvanic effects and promote localized corrosion [57,59,60,61,62,63], whereas Si promotes ε-martensite formation and shape-memory behavior in Fe–Mn–Si systems, while also affecting Young’s modulus, corrosion response, and phase transformation behavior [64,65,66,67,68,69]. Therefore, Mn, C, and Si do not simply act as strengthening additions; rather, they control the balance between phase constitution, deformation mechanism, magnetic compatibility, and degradation behavior in Fe-based biodegradable alloys [40,51,58,59,60,61,64,66].

A second group of alloying elements is primarily introduced to accelerate degradation. Noble elements such as Pd, Ag, Au, Pt, and Cu promote micro-galvanic corrosion through the formation of cathodic second phases [52,70,71,72,73,74,75,76,77,78,79,80,81,82]. Among these, Pd is particularly effective, increasing degradation rates by nearly an order of magnitude while retaining high mechanical strength [70,71,72,73]. Ag and Cu can also accelerate degradation and provide antibacterial activity, although their effectiveness depends on particle distribution, ion release, and processing route [41,74,75,76,77,78,83,84,85,86,87]. Ca and Mg are less commonly used but are attractive for bone applications because of their osteogenic potential; however, their limited solubility in Fe creates challenges related to segregation, secondary phases, and localized corrosion [88,89,90,91]. Overall, future Fe alloy design is moving toward multi-element systems that combine degradation control, mechanical integrity, antibacterial response, magnetic compatibility, and radiopacity [12,52,79,80,81,82,92,93,94]. Representative Fe-based bioresorbable alloy systems and their main alloying strategies, processing routes, reported effects, and remaining limitations are summarized in Table 6.

This comparison highlights that no single Fe-based alloying strategy simultaneously resolves all key requirements, including controlled and sufficiently uniform degradation, adequate mechanical integrity, magnetic compatibility, and acceptable biological response [12,49,50,53].

#### 3.2.2. Fe Thermomechanical Processing

Thermomechanical processing is used not only to improve strength and ductility but also to modify corrosion behavior, phase stability, and microstructural evolution. Hot rolling, cold rolling, rolling plus heat treatment, forging, swaging, wire/tube drawing, SPD, and hot extrusion refine grains, increase defect density, modify texture, and redistribute phases [12,64,114,115]. Hot rolling has been widely applied to Fe–Mn, Fe–Mn–Si, Fe–Mn–C, and Fe–Mn–C–Ag alloys at 600–1100 °C, improving microstructural homogeneity and changing the γ-austenite/ε-martensite balance [63,65,92,97,98,116]. Cold rolling increases strength through work hardening but may reduce ductility; its effect on corrosion depends on alloy chemistry, strain level, and annealing [57,59,64,92].

Rolling combined with annealing, recrystallization, solution treatment, or quenching is particularly important in Fe–Mn–Si systems because it controls grain size, residual stress, γ ↔ ε martensitic transformation, Young’s modulus, and electrochemical response [65,66,96,98,114]. Bulk compressive processes such as forging, rotary swaging, radial-shear rolling, and hot compression improve homogeneity and can generate deformation bands, twins, and phase changes that affect both mechanical properties and degradation [41,51,67,114]. Wire and tube drawing are essential for Fe–Mn-based stents and neurovascular devices, as they produce fine geometries with high radial strength, although intermediate annealing is often required to prevent excessive loss of ductility [58,62,93,94,95]. SPD methods such as ECAP and HPT can generate ultrafine or nanostructured Fe alloys, but corrosion behavior does not always increase with grain refinement because phase transformations may reduce micro-galvanic activity [68,69,99]. Hot extrusion remains less explored, mainly in Fe–Mn–Ca alloys, and systematic studies on extrusion temperature, ratio, and speed are still lacking [100]. The main thermomechanical and manufacturing routes applied to Fe-based biodegradable alloys are summarized in Table 7. These processing routes not only improve mechanical properties, but also modify phase stability, defect density, texture, secondary-phase distribution, and corrosion response [12,64,88,114,115].

Overall, thermomechanical processing provides an important route for improving the mechanical performance of Fe-based biodegradable alloys; however, its effect on degradation is not straightforward. Grain refinement, increased defect density, and phase redistribution may enhance corrosion activity in some systems, whereas processing-induced phase transformations may reduce micro-galvanic effects or change the stability of the surface film. Therefore, processing parameters must be optimized together with alloy composition rather than considered as an independent variable [12,64,66,68,99].

#### 3.2.3. Fe Porous Architectures and Manufacturing Approaches

Porous Fe-based structures provide an extrinsic route to accelerate degradation by increasing exposed surface area, improving electrolyte penetration, and reducing stiffness toward values closer to bone. This approach is especially relevant because bulk Fe alloys often remain too corrosion-resistant for biodegradable applications. Porous Fe-based scaffolds have been fabricated using several processing routes, including powder metallurgy with space holders, sponge replication, LPBF/SLM, binder jetting, extrusion-based 3D printing/direct ink writing (DIW), and hybrid 3D printing followed by sintering. Among these methods, powder metallurgy and space-holder techniques provide relatively simple control over porosity and pore size, whereas LPBF/SLM enables more precise control of ordered lattice architectures. Binder jetting is also relevant because it can fabricate complex porous Fe-based scaffolds without direct laser melting; however, its final properties are strongly affected by debinding, sintering shrinkage, residual porosity, and post-sintering microstructure. In contrast, extrusion-based 3D printing/DIW has recently attracted attention because it enables the fabrication of highly porous Fe, Fe–Mn, and Fe-based bioactive composite scaffolds with controlled architecture and improved biological functionality. Therefore, this section emphasizes the routes for which more complete relationships among processing route, scaffold architecture, degradation behavior, mechanical response, and biological performance have been reported [12,24,50,53,110,111,112,117]. However, increasing porosity reduces load-bearing capacity and fatigue resistance, making the balance between degradation rate, mechanical integrity, and biological performance critical [20,50,53,101,102,112,118]. Porous architectures also promote tissue integration by enabling cell migration, vascularization, and nutrient transport and new tissue ingrowth [20,50,53], as schematically shown in Figure 4.

Powder metallurgy and space-holder techniques are widely used conventional routes because they allow control of pore size and porosity through powder characteristics, space-holder content, compaction pressure, and sintering parameters [16,53,102,104]. Porosity levels around 30–50% can maintain properties suitable for selected orthopedic applications, and bioactive phases such as hydroxyapatite can be incorporated to improve biological response [16,102,105]. Sponge replica methods produce highly interconnected open-cell structures; Fe–Mn scaffolds with ~85% porosity and 375–500 μm pores have shown properties comparable to cancellous bone, although load-bearing capacity remains limited [106,119,120,121] LPBF/SLM enables digital control of pore architecture, strut thickness, and porosity, and Fe–35Mn scaffolds have shown promising biodegradation, biocompatibility, and mechanical performance for porous load-bearing bone scaffold applications [53,107,108,109]. However, defects, residual stress, surface roughness, and elemental evaporation must be controlled [107,108,109]. Extrusion-based 3D printing/DIW offers a lower-cost alternative and has enabled Fe–Mn and FeMn–akermanite scaffolds with improved biodegradability, osteogenic response, and MRI compatibility [24,110,111,112,113]. Hybrid approaches combining AM, powder metallurgy, and sintering can create hierarchical porous structures with CAD-designed macropores and sintering-induced micropores [103,122,123,124]. Overall, the most promising route for Fe-based biodegradable implants is the integration of alloy design, thermomechanical processing, and controlled porous architecture to achieve degradation synchronized with tissue healing while maintaining temporary mechanical support [12,20,24,50,53,107].

## 4. Mg-Based Alloys

### 4.1. Comparison to Fe and Clinical Motivation

Magnesium-based alloys have attracted considerable attention as biodegradable implants because they address several limitations of permanent metallic biomaterials and Fe-based biodegradable systems. Unlike stainless steels and titanium alloys, Mg exhibits an elastic modulus of approximately 40–45 GPa, much closer to cortical bone, reducing stress shielding and promoting more natural load transfer during healing. Compared with Fe-based alloys, Mg offers superior biomechanical compatibility and a more suitable degradation timeframe. However, Mg alloys generally possess lower mechanical strength and significantly higher corrosion rates, making degradation control the primary challenge for clinical application [5,6,13,125].

### 4.2. Challenges

The main limitation of Mg alloys is their high chemical reactivity in physiological environments. Corrosion leads to the formation of Mg(OH)_2_ and the evolution of hydrogen gas, often resulting in degradation rates that exceed tissue healing requirements. Rapid corrosion can generate gas cavities around the implant, affecting tissue regeneration, implant stability, and, in severe cases, causing local tissue damage. Simultaneously, the accelerated loss of material may lead to premature reduction in mechanical integrity and implant failure before healing is complete. In addition, Mg degradation causes local alkalization through Mg(OH)_2_ formation and OH^−^ accumulation; however, the magnitude of pH increase strongly depends on the test solution, buffering capacity, ion composition, and solution volume/surface area ratio. For example, Lamaka et al. measured the local pH 10–50 μm above Mg alloy surfaces in Hank’s solution and reported near-surface pH values of approximately 9.9–10.5 in simple Hank’s solution, whereas Ca^2+^-modified Hank’s solution maintained lower values of about 7.8–8.5 owing to the formation of calcium-phosphate/carbonate surface layers. Yang and Zhang further showed that Mg alloy corrosion in Hank’s solution and simulated blood plasma is affected by chloride concentration, Ca^2+^/PO_4_^3−^ content, and solution volume/surface area ratio, confirming that pH evolution and corrosion kinetics must be interpreted together with the selected immersion medium and testing geometry. Such localized alkalization may impair cellular activity and tissue compatibility if buffering and corrosion-product formation are insufficient [46,47,126].

Beyond corrosion, Mg alloys exhibit relatively low fatigue resistance and are highly sensitive to microstructural heterogeneities, which can further promote localized degradation. Consequently, controlling corrosion kinetics while maintaining adequate mechanical performance remains the key challenge limiting the broader clinical adoption of Mg-based biodegradable implants [5,13,14,127]. It should also be noted that the corrosion kinetics of Mg-based biodegradable alloys cannot be fully described by a single corrosion-rate value, because the degradation process is strongly time-dependent and affected by the selected testing method. Electrochemical techniques such as OCP, potentiodynamic polarization, and EIS mainly reflect early-stage surface reactions, passive-film stability, and charge-transfer resistance, whereas hydrogen-evolution, immersion, and weight-loss measurements provide longer-term information on dissolution, corrosion-product accumulation, and degradation progression. Therefore, corrosion-rate data for Mg alloys should be interpreted together with test duration, solution composition, pH evolution, solution renewal, and the surface-area/solution-volume ratio [44,45,47].

From an electrochemical-kinetics perspective, Mg corrosion is mainly governed by rapid anodic dissolution of Mg and cathodic hydrogen evolution, whereas the temporary formation and breakdown of Mg(OH)_2_-based corrosion layers control the transition from early-stage surface activation to longer-term degradation. Potentiodynamic polarization can provide useful parameters such as corrosion potential, corrosion current density, and apparent corrosion rate; however, for Mg alloys these values should be interpreted cautiously because negative difference effects, film rupture, hydrogen evolution, and localized attack may distort Tafel extrapolation. EIS is therefore particularly useful for evaluating the evolution of surface-film resistance, charge-transfer resistance, and diffusion-related processes during immersion. A larger capacitive loop and higher polarization resistance generally indicate improved short-term corrosion resistance, whereas the appearance of inductive loops or decreasing impedance at low frequency often reflects film instability, adsorption/desorption of intermediate species, or localized corrosion activity. Thus, polarization and EIS results should be correlated with immersion observations, hydrogen evolution, pH variation, and corrosion-product characterization rather than used as isolated indicators of Mg degradation behavior [44,45,128,129,130].

### 4.3. Mg Development: Alloying, Bulk Processing, and Porous Architectures

Following the general development strategy illustrated in Figure 4, research on Mg-based biodegradable materials has focused on two parallel directions: optimization of dense bulk alloys through alloy design and thermomechanical processing and the development of porous architectures to improve mechanical compatibility and regulate degradation behavior. While bulk alloy development primarily aims to reduce the intrinsically high corrosion rate of magnesium, porous structures exploit geometric design to simultaneously promote biological integration and control degradation kinetics. These two approaches are increasingly combined to achieve a balanced relationship between mechanical integrity, corrosion resistance, and tissue regeneration [13,14,21,125].

#### 4.3.1. Development of Bulk Mg-Based Alloys

In bulk form, Mg alloys are primarily optimized through alloying and thermomechanical processing to improve mechanical performance while reducing corrosion rates [13,14,125,127]. Alloying additions such as Zn, Ca, Mn, Zr, Sr, and rare earth (RE) elements play a central role in tailoring microstructure and electrochemical behavior. Ca and Zn are particularly attractive because they are naturally present in the human body and contribute to bone metabolism. Controlled additions of Ca improve biocompatibility and osteogenic potential; however, excessive Ca promotes the formation of Mg_2_Ca intermetallic phases that can accelerate localized galvanic corrosion and reduce ductility [131,132,133,134,135]. Similarly, Zn contributes to solid-solution strengthening, grain refinement, and stabilization of corrosion products, although excessive Zn may increase micro-galvanic activity through the formation of Zn-rich secondary phases [125,136,137,138,139,140].

Rare earth elements such as Y, Nd, and Gd are among the most effective alloying additions for biodegradable Mg alloys. These elements refine grains, modify crystallographic texture, improve mechanical strength, and contribute to the formation of more stable surface films, thereby reducing corrosion rates [141,142,143,144,145,146]. Additional alloying elements including Sr, Zr, Ag, Cu, Li, Si, and Sn have also been investigated to improve degradation behavior, antibacterial performance, and mechanical properties, although their use requires careful control to avoid excessive galvanic corrosion or potential biocompatibility concerns [125,147,148,149,150,151,152,153,154,155,156,157].

Beyond alloy composition, thermomechanical processing is one of the most effective strategies for microstructural optimization. Hot rolling, hot extrusion, cold rolling followed by annealing, forging, multidirectional forging (MDF), equal-channel angular pressing (ECAP), and high-pressure torsion (HPT) have all been employed to refine grain size, redistribute secondary phases, and reduce casting defects. Dynamic recrystallization during rolling and extrusion produces finer and more homogeneous microstructures, improving both mechanical strength and corrosion resistance through grain refinement, reduced segregation, and a more uniform distribution of secondary phases. In Mg-based biodegradable alloys, hot extrusion is particularly important because it converts coarse cast structures into refined recrystallized microstructures, breaks up brittle intermetallic networks, and redistributes cathodic second phases that otherwise act as local galvanic sites. Shiri et al. reviewed the role of extrusion parameters in biodegradable Mg alloys and showed that extrusion temperature, extrusion ratio, and extrusion speed strongly influence degradation behavior through their effects on dynamic recrystallization, basal texture, grain size, and secondary-phase distribution. For example, in extruded Mg–Y–Zn–Zr alloys containing W and I phases, increasing the extrusion temperature from 250 °C to 300 °C improved corrosion resistance because of more complete dynamic recrystallization, grain refinement, and a more homogeneous distribution of broken second phases; however, further increasing the temperature to 350 °C reduced the benefit because grain coarsening and second-phase effects became more pronounced. More specifically, Wu et al. reported that Mg–2Y–1Zn–0.6Zr alloy extruded at 300 °C exhibited the best corrosion resistance in SBF, with a weight-loss corrosion rate of 0.4601 mm/year, whereas the corrosion-rate sequence followed E250 °C > E350 °C > E300 °C. This behavior was attributed to sufficient dynamic recrystallization and finer, more uniform grains at 300 °C, while undissolved W and I phases or unrecrystallized regions at lower extrusion temperature, and grain coarsening at higher extrusion temperature, promoted stronger micro-galvanic corrosion [158]. Similar observations have been reported in Mg–Zn–Ca–Zr systems, where optimized extrusion temperatures produced lower corrosion rates and more uniform degradation, whereas non-optimized extrusion conditions or excessive secondary-phase precipitation could increase localized corrosion. Therefore, the beneficial effect of extrusion on Mg corrosion is not universal; it depends on the balance between grain refinement, texture evolution, residual strain, and the size, chemistry, and distribution of intermetallic phases [14,158,159,160,161,162,163,164].

The effect of heat treatment on Mg corrosion should be interpreted according to alloy family rather than treated as a universal response. In Mg–Al systems, solution treatment can dissolve or redistribute β-Mg_17_Al_12_ phases and reduce micro-galvanic coupling when the secondary phase network is discontinuous; however, inappropriate aging may reintroduce cathodic precipitates and increase localized corrosion. In Mg–Zn and Mg–Zn–Zr alloys, T4 treatment may improve corrosion resistance by dissolving Zn-rich secondary phases and homogenizing solute distribution, whereas T6 aging can either improve strength through precipitation hardening or reduce corrosion resistance if precipitates become coarse, continuous, or strongly cathodic relative to the Mg matrix. In Mg–Y–Nd–Zr and other Mg–RE systems, heat treatment modifies RE-rich β′ and β1 nanophases, grain-boundary precipitates, and surface-film stability; therefore, T6 treatment may improve passivation and mechanical strength in some WE43-type alloys but may also promote localized attack when precipitates or eutectic remnants are non-uniformly distributed. Thus, heat treatment should be optimized separately for each Mg alloy family because corrosion resistance depends on the combined effects of solute homogenization, precipitate morphology, grain size, and cathodic second-phase distribution [165,166,167]. The alloy-family-dependent effects of heat treatment on the microstructure and corrosion behavior of representative biodegradable Mg alloys are summarized in Table 8. Representative studies further show that the corrosion response after heat treatment is mainly governed by the morphology, continuity, and electrochemical role of secondary phases rather than by the heat-treatment condition alone. In Mg–Al alloys, solution treatment can reduce micro-galvanic corrosion by dissolving or disrupting continuous β-Mg17Al12 networks; however, subsequent aging may reintroduce cathodic β-Mg17Al12 precipitates and increase localized corrosion if the precipitates become coarse or continuous. In Mg–Zn and Mg–Zn–Zr alloys, T4 treatment can improve corrosion resistance by dissolving Zn-rich phases and homogenizing solute distribution, whereas T6 aging may either improve mechanical strength through precipitation hardening or reduce corrosion resistance when Zn-rich precipitates form strong local cathodic sites. In Mg–Y–Nd–Zr and WE43-type alloys, heat treatment affects RE-rich precipitates, especially β′ and β1 nanophases, as well as grain-boundary particles and surface-film stability. Therefore, the beneficial or detrimental effect of heat treatment depends on whether secondary phases become fine, discontinuous, and uniformly distributed or coarse, continuous, and electrochemically active relative to the Mg matrix [165,166,167].

This comparison shows that heat treatment cannot be evaluated independently of alloy chemistry. In Mg alloys, the same treatment condition may improve corrosion resistance in one alloy family by dissolving harmful secondary phases but decrease corrosion resistance in another system by producing coarse or continuous cathodic precipitates. Therefore, the corrosion response of heat-treated Mg alloys should be interpreted together with phase constitution, precipitate distribution, electrochemical potential differences, and the stability of the corrosion-product layer [165,166,167]. Overall, bulk processing strategies for Mg-based biodegradable alloys should be evaluated through the combined effects of grain refinement, crystallographic texture, dynamic recrystallization, residual strain, and secondary-phase redistribution. Although extrusion, rolling, and heat treatment can substantially improve strength and ductility, their influence on corrosion is alloy-dependent and sometimes contradictory. Grain refinement and solute homogenization may reduce micro-galvanic heterogeneity and promote more uniform degradation, whereas strong basal texture, residual deformation defects, or continuous cathodic intermetallic phases may accelerate localized attack. Therefore, future development of bulk Mg-based implants should move from single-parameter optimization toward integrated control of processing route, alloy chemistry, microstructure, corrosion kinetics, and mechanical-integrity retention during degradation [14,158,164,165,167].

Processing-induced crystallographic texture also plays a critical role. Magnesium’s hexagonal close-packed (HCP) structure makes corrosion behavior strongly orientation-dependent. Consequently, texture control has become as important as grain refinement, since strong basal textures may induce mechanical and corrosion anisotropy that can compromise implant reliability [168,169,170,171]. Similarly, forging and MDF processing routes improve strength and corrosion resistance by refining grains and modifying texture, although excessive deformation can increase residual stresses and promote localized corrosion [172,173,174,175,176].

Severe plastic deformation techniques such as ECAP and HPT represent advanced processing routes capable of producing ultrafine-grained Mg alloys. These methods significantly enhance strength through grain refinement and may improve corrosion resistance when microstructural homogeneity is maintained. However, excessive residual stresses and heterogeneous distributions of secondary phases may offset some of these benefits [127,177,178,179,180,181,182,183].

Surface modification strategies are commonly employed alongside alloy and processing optimization. Calcium-phosphate coatings, hydroxyapatite layers, polymeric coatings, plasma electrolytic oxidation (PEO), fluoride treatments, and oxide films act as temporary barriers that reduce the initial corrosion rate and preserve mechanical integrity during early healing stages. Nevertheless, their long-term effectiveness depends on coating adhesion, degradation kinetics, and interactions with the underlying substrate [25,26,27,184,185].

Despite these advances, bulk Mg alloys continue to face challenges associated with rapid degradation, hydrogen evolution, localized alkalization, and premature mechanical failure in aggressive physiological environments. Consequently, structural approaches based on engineered porosity have emerged as a complementary strategy [5,6,21,186].

#### 4.3.2. Development of Porous Mg-Based Structures

Porous Mg-based structures represent an alternative approach to overcoming the limitations of bulk materials by exploiting architectural design to tailor mechanical properties, degradation behavior, and biological performance. The introduction of interconnected porosity facilitates cell migration, vascularization, nutrient transport, and bone ingrowth while simultaneously reducing the elastic modulus toward values closer to those of cancellous bone, thereby mitigating stress shielding effects [186,187].

Unlike Fe-based systems, where porosity is primarily introduced to accelerate degradation, Mg scaffolds require careful architectural design because increased surface area generally accelerates corrosion and hydrogen evolution. Therefore, scaffold design must balance porosity, pore size, interconnectivity, wall thickness, mechanical strength, and degradation rate [21,186,187].

Powder metallurgy combined with space-holder techniques remains one of the most widely used fabrication routes. In these methods, temporary pore-forming agents such as ammonium bicarbonate, urea, or salt particles are removed after compaction and sintering, producing interconnected pore networks with controllable pore morphology [17,18,186,188,189,190]. These techniques enable tuning of pore size and porosity, although challenges remain related to powder oxidation, incomplete sintering, residual MgO formation, and insufficient control of local corrosion processes [17,18,186,190].

Additive manufacturing has emerged as one of the most promising technologies for biodegradable Mg scaffolds because it enables precise control of pore geometry, unit-cell architecture, strut thickness, and spatial porosity distribution [21,185,191,192,193,194,195]. Technologies such as laser powder bed fusion (LPBF) and selective laser melting (SLM) have demonstrated the ability to fabricate highly ordered porous structures with mechanical properties approaching those of trabecular bone. However, magnesium processing remains challenging due to its high vapor pressure, oxidation tendency, flammability, and susceptibility to powder spattering and evaporation during laser processing [21,185,194].

Recent studies have demonstrated that scaffold architecture can significantly influence degradation behavior. Parameters such as pore size, pore morphology, strut thickness, and surface roughness directly affect corrosion kinetics, mechanical stability, and osteogenic response [185,192,195]. Larger interconnected pores generally improve bone ingrowth and vascularization, whereas excessively high porosity may lead to rapid loss of mechanical integrity and accelerated degradation [189,192].

Alternative fabrication routes include infiltration casting, sacrificial-template methods, replica techniques, and metallic foaming approaches [27,196,197,198,199]. These methods can generate highly interconnected open-cell structures and often provide better metallurgical continuity than powder-based routes. However, they require careful control of melt infiltration, template removal, and oxidation during processing. Residual contaminants from templates may significantly influence corrosion behavior and biocompatibility [196,197,198]. The main fabrication routes for porous Mg-based biodegradable scaffolds and their architecture-related advantages and limitations are summarized in Table 9.

This comparison emphasizes that porous Mg scaffold design is more complex than simply increasing [17,18,27,185,186,191,192,193,196,197,198,199] porosity. Although interconnected pores improve cell migration, vascularization, nutrient transport, and bone ingrowth, they also increase the exposed surface area and may accelerate Mg dissolution, hydrogen evolution, and early mechanical loss. Therefore, the design of porous Mg-based implants requires simultaneous optimization of pore size, porosity, interconnectivity, strut thickness, surface condition, coating stability, and alloy composition. In this regard, graded and hierarchical architectures are particularly attractive because dense regions can preserve load-bearing capacity, whereas porous regions can promote tissue integration and controlled degradation [17,18,27,185,186,191,192,193,196,197,198,199].

To address the conflicting requirements of mechanical support and biological performance, recent developments increasingly focus on graded and hierarchical architectures. Dense regions provide load-bearing capacity, whereas porous regions promote tissue integration and controlled degradation. Furthermore, combining porous architectures with surface coatings such as hydroxyapatite, calcium-phosphate, fluoride, or oxide layers has shown considerable potential for mitigating the accelerated corrosion typically associated with highly porous Mg structures [27,185,192,199].

Overall, the integration of alloy design, thermomechanical processing, advanced manufacturing technologies, and surface engineering is driving the development of next-generation biodegradable Mg implants. Future research is expected to focus on hierarchical scaffold architectures, patient-specific additive manufacturing, and multiscale strategies that simultaneously optimize mechanical performance, degradation kinetics, and biological functionality [5,6,21,53,185,192].

## 5. Zn-Based Alloys

### 5.1. Comparison to Fe and Mg

Zinc-based alloys have emerged as a promising alternative to both Mg- and Fe-based biodegradable metals because they exhibit an intermediate degradation rate that is closer to the clinically desired window. Unlike Mg, Zn does not generate hydrogen gas during corrosion, while compared with Fe it degrades more rapidly, reducing the risk of long-term implant persistence. Mechanically, Zn alloys occupy an intermediate position between Mg and Fe, combining moderate strength, ductility, and stiffness, making them attractive for orthopedic and cardiovascular applications [7,9,200,201].

### 5.2. Challenges

Despite these advantages, pure Zn generally suffers from relatively low mechanical strength in the as-cast condition, often remaining below the levels required for demanding load-bearing applications. Its ductility may also be restricted in the as-cast state because of coarse grains, segregation, and limited activation of deformation mechanisms. However, this limitation should not be considered intrinsic to Zn, because extrusion, rolling, drawing, and severe plastic deformation can substantially improve ductility, strength, and mechanical reliability by refining grains, modifying texture, and redistributing second phases. In addition, Zn corrosion should not be regarded as intrinsically uniform. Alloying-induced second phases, eutectic constituents, crystallographic texture, additively manufactured defects, and local micro-galvanic couples can promote heterogeneous or localized corrosion. Therefore, the performance of Zn alloys is highly processing-sensitive and depends on the combined control of alloy chemistry, grain size, texture, secondary-phase morphology, and manufacturing route [9,15,109,202,203].

### 5.3. Development of Zn-Based Biodegradable Metals: From Bulk Alloys to Porous Architectures

#### 5.3.1. Alloy Design of Bulk Zn-Based Biodegradable Alloys

Alloying is one of the main strategies used to improve the limited mechanical properties of pure Zn while preserving its favorable degradation behavior. Different alloying elements are added to enhance strength, ductility, corrosion resistance, biocompatibility, antibacterial activity, and bone-regeneration potential [9,200,201,204,205].

Zn–Mg alloys are among the most successful and widely studied Zn-based systems. Mg is biocompatible, promotes grain refinement, improves cell adhesion, and can bring the mechanical response of Zn alloys closer to that of natural bone [204,206,207]. However, Mg forms brittle Mg_2_Zn_11_ and MgZn_2_ phases, which increase strength through precipitation hardening but may reduce ductility and promote localized galvanic corrosion [206,207,208]. Therefore, Mg content and intermetallic morphology must be carefully controlled. Processing routes such as extrusion and ECAP can fragment and homogenize these phases, improving strength, ductility, and corrosion uniformity [204,207,209].

Li is one of the strongest alloying additions for Zn. It increases strength and hardness while reducing elastic modulus, which is beneficial for bone-related applications [10,204,210]. Its strengthening effect is mainly associated with the β-LiZn_4_ phase, which restricts dislocation motion. However, excessive Li may cause brittleness [10,210]. Thermomechanical processing, such as extrusion and rolling, refines the microstructure and secondary phases, while additions such as Mn can further improve the strength–ductility balance [211,212,213]. Li-containing corrosion products such as LiOH and Li_2_CO_3_ may form a relatively protective layer in SBF, although corrosion behavior strongly depends on the testing medium [184,214].

Mn is attractive due to its biocompatibility, low toxicity, and role in bone metabolism [203,204,215]. It contributes to grain refinement, solid-solution strengthening, twinning, texture modification, and improved plastic deformation [203,204,215]. At low contents, MnZn_13_ intermetallics may be beneficial, while excessive Mn can increase localized corrosion and stress concentration [200,215]. In some systems, Mn produces exceptional ductility; for example, Zn–0.8Li–0.8Mn showed elongation of about 103% [10]. In extruded Zn–0.2Mg, Mn promoted grain rotation and increased the Schmid factor for non-basal slip, improving plasticity [216].

Cu improves strength, antibacterial activity, bone growth, and angiogenesis [204,215,217]. Its strengthening effect is mainly related to solid-solution strengthening, grain refinement, and hard intermetallic phases such as ε-CuZn_5_/CuZn_4_ [10]. However, coarse Cu-rich phases can reduce ductility and intensify localized corrosion due to galvanic coupling [204,217]. Thermomechanical processing can refine these phases and improve corrosion uniformity. In Zn–Cu alloys, refined precipitates may activate phase-boundary sliding at Zn/CuZn_4_ interfaces, enabling room-temperature superplasticity [217,218,219].

Ca is especially relevant for bone implants because of its high bioactivity, low toxicity, and positive role in bone regeneration [200,206]. However, its very low solubility in Zn leads to the formation of coarse and brittle CaZn_13_ intermetallics, which reduce ductility and promote localized galvanic corrosion [200,203]. Therefore, controlling Ca-containing precipitates is essential. Recent studies show that ultrasonic treatment, hot rolling, and optimized casting can refine CaZn_13_ particles and significantly improve ductility; for example, Zn–0.3Ca achieved 49% elongation after strong refinement of CaZn_13_ particles and Zn grains [220,221].

Sr stimulates osteogenesis, mineralization, and bioactivity [200,222]. Like Ca, it forms SrZn_13_ intermetallics due to limited solubility in Zn but generally causes less brittleness [200,204]. Thermomechanical processing can refine SrZn_13_ phases and improve the balance between strength, ductility, and degradation. Low Sr contents may promote more uniform corrosion, while excessive Sr can increase intermetallic content and microgalvanic corrosion [200,222]. A Zn–1Sr alloy showed compressive superplasticity and a yield strength of about 340 MPa [223].

Ag provides solid-solution strengthening and antibacterial functionality [200,204,224]. It forms ε-AgZn_3_ intermetallics, improving creep resistance, hardness, and mechanical performance at body temperature [224]. Ag may also increase strain hardening, which is important for stents because insufficient strain hardening can cause recoil or deformation instability [225]. However, Ag-rich phases may promote localized or pitting corrosion because the Zn matrix corrodes preferentially relative to Ag-rich regions [200,224]. Under cold working, Zn/AgZn_3_ interfaces may activate phase-boundary sliding, leading to strain softening and even room-temperature superplasticity [15,224,226].

Rare-earth elements such as Y, Nd, Er, Ho, and Dy have recently gained attention in biodegradable Zn alloys. They usually have low solubility in Zn and act through intermetallic formation, grain refinement, grain-boundary pinning, dynamic recrystallization stimulation, texture modification, and corrosion control [227,228,229,230]. Among various rare-earth elements, Y, Ho, and Lu showed the strongest improvements in mechanical properties and biocompatibility, while Eu, Gd, and Dy showed weaker effects [228]. However, their influence on corrosion is dual: they may improve corrosion resistance by refining grains and forming denser corrosion layers, but Zn–REE intermetallics may also act as microgalvanic corrosion sites. Therefore, the morphology, size, and distribution of rare-earth phases must be carefully controlled [228,231,232].

#### 5.3.2. Thermomechanical Processing of Bulk Zn-Based Alloys

Hot extrusion, hot rolling, cold rolling, ECAP, hydrostatic extrusion, and HPT are the main thermomechanical routes applied to biodegradable Zn-based alloys. These processes aim to promote dynamic recrystallization and grain refinement, fragment intermetallic phases, homogenize precipitate distribution, modify crystallographic texture, and improve corrosion behavior [15,200,204,233]. Their effect on corrosion can be both positive and negative. Beneficial effects include the formation of a more uniform corrosion layer and fewer galvanic cells due to grain refinement, homogeneous phase distribution, and elimination of coarse eutectic structures [200,204,207]. However, increased dislocation density, grain-boundary area, and fragmented phases may also increase electrochemical activity and initial corrosion rate [15,204]. Overall, thermomechanical processing generally makes corrosion more uniform, even if the early corrosion rate slightly increases [200,204].

Hot extrusion is one of the most effective processes for simultaneously improving strength and ductility in Zn-based alloys. Through dynamic recrystallization, it reduces grain size, breaks brittle phases, and distributes them more uniformly, increasing yield strength, ultimate tensile strength, and elongation [10,204,206]. In Zn–Mg and Zn–Li systems, extrusion and rolling can increase tensile strength above 400 MPa while maintaining useful ductility [10,213]. In Zn–Mg alloys, typical post-extrusion tensile strength is 220–280 MPa with elongation of 10–20%, depending on composition, extrusion ratio, temperature, and final microstructure [206,207,209]. Reported extrusion temperatures range from 100 to 320 °C, with 200–300 °C being the most common and optimal range because it activates dynamic recrystallization, reduces flow stress, prevents hot cracking, limits grain growth, and improves both strength and ductility [15,228,234,235,236,237,238,239,240,241,242,243,244,245]. Extrusion ratios from 1:9 to 1:100 have been reported [15,227,228,234,235,236,237,238,239,241,242,243,244,245]. Extrusion speed has been less systematically studied, but typical values range from 0.1 to 20 mm/s. Nienaber et al. showed that increasing extrusion speed can increase flow heterogeneity during Zn-based wire processing [246].

Hot rolling improves ductility, reduces porosity, orients the microstructure, and enhances plastic deformation in Zn alloys [203,247]. In Zn–Mn alloys, elongation can increase from below 1% in the as-cast state to 35–40% after hot rolling [201,203]. Similar ductility improvements have been reported for Zn–Cu and Zn–Li alloys [201,213,247]. The reported temperature range is 210–350 °C, while 220–350 °C is considered optimal due to dynamic recrystallization, texture improvement, reduced edge cracking, and enhanced ductility [218,230,232,240,247,248,249,250]. Because Zn alloys have an HCP crystal structure, reduction per pass must be carefully controlled; excessive reduction may cause cracking, strain localization, texture intensification, and fracture of intermetallic particles. Reported reductions range from 0.5 mm/pass to 25–33% per pass [251], while total reductions are commonly 50–90%, with 50–75% considered optimal. Small reductions per pass promote gradual recrystallization, weaker texture, fewer edge cracks, and more uniform corrosion [218,227,230,232,247,248,250,252].

ECAP is one of the most effective severe plastic deformation methods for producing ultrafine-grained Zn alloys, leading to strong strengthening but sometimes reduced ductility [15,253,254]. In Zn–3Mg, two ECAP passes increased tensile strength from about 84 MPa in the as-cast state to about 220 MPa [253]. In multicomponent alloys, hybrid processing can produce tensile strengths of 350–420 MPa depending on composition, number of passes, and temperature [212,255]. ECAP temperatures between 50 and 200 °C have been reported, while 100–200 °C is most common because it prevents cracking, activates dynamic recrystallization, promotes grain refinement, and limits grain growth [15,253,254]. Up to eight ECAP passes have been reported for Zn alloys [15,253]. Recent work on Zn–Li alloys shows that controlled ECAP processing can increase tensile strength above 400 MPa [255]. Hybrid processing, especially hot extrusion followed by ECAP, has become increasingly important because it combines strong grain refinement with eutectic fragmentation, texture modification, and improved mechanical and corrosion behavior [212].

Cold rolling increases dislocation density, promotes nanoscale precipitation, produces ultrafine grains, and enhances strength in Zn alloys [213,217,247]. In Zn–Cu alloys, cold rolling can form nanoscale ε-CuZn_4_ precipitates and, under certain conditions, induce superplastic behavior [217]. Due to the low processing temperature and HCP structure of Zn, reduction per pass and total reduction must be lower than in hot processing. Typical reductions are 3–10% per pass and 10–50% total reduction, with 20–40% considered optimal [201,247].

HPT is among the most severe SPD processes. By imposing extremely high strain, it produces ultrafine-grained or nanostructured microstructures, homogenizes intermetallic phase distribution, and strongly increases strength [15,254]. In some multicomponent Zn alloys, tensile strength close to 500 MPa has been reported after HPT, together with improved microstructural uniformity and corrosion behavior [245,254].

#### 5.3.3. Additive Manufacturing of Dense Zn-Based Materials

As illustrated in Figure 4, the development of additively manufactured biodegradable metals also follows the two complementary pathways of dense bulk components and porous scaffolds. In Zn-based systems, however, particular attention has been devoted to optimizing LPBF processing conditions because of the low melting and boiling temperatures of Zn, followed by alloy design, microstructure engineering, and scaffold architecture optimization [22,247,256,257]. Early studies on LPBF-processed Zn primarily focused on improving densification and establishing stable processing windows. Montani et al. [256] were the first to demonstrate the feasibility of fabricating biodegradable Zn using LPBF; however, the relative density of the fabricated samples reached only 88%, mainly due to severe Zn evaporation during processing. To alleviate vapor accumulation and improve process stability, Demir et al. [257] and Lietaert et al. [258] optimized the processing environment and gas-flow conditions, enabling the fabrication of pure Zn components with relative densities exceeding 98%, although within a relatively narrow processing window. Further improvements were achieved by Wen et al. [259], who developed a dedicated gas-circulation system to suppress smoke accumulation and spatter formation, resulting in a relative density of 99.9%. The optimized pure Zn exhibited an ultimate tensile strength of approximately 122 MPa and an elongation of ~8%, demonstrating the feasibility of producing near fully dense Zn components by LPBF. Nevertheless, these mechanical properties remained insufficient for many load-bearing biomedical applications [258].

Beyond densification, several studies investigated the influence of LPBF processing conditions on the microstructure and performance of pure Zn. Qin et al. [260] showed that grain refinement induced by higher scanning speeds improved both strength and ductility, while build orientation introduced pronounced mechanical anisotropy. Similarly, Dong et al. [261] reported tensile strengths exceeding 120 MPa together with elongations approaching 14%, highlighting the beneficial effects of the refined non-equilibrium microstructures generated during rapid solidification. Although LPBF significantly improved the mechanical properties of pure Zn compared with its casting, the achieved strength levels still fell considerably below the requirements for biodegradable fixation devices and vascular implants [260,261].

Consequently, research efforts increasingly shifted toward alloy design and microstructural engineering to overcome the intrinsic strength limitations of pure Zn while maintaining its favorable biodegradation characteristics. Among the various alloying strategies explored, Mg was one of the earliest and most extensively investigated alloying elements because of its strong strengthening effect and good biocompatibility. The development of LPBF-fabricated Zn alloys has since expanded to include Ag, Cu, Li, Mn, and multicomponent alloy systems, aiming to achieve an improved balance between strength, ductility, degradation behavior, and biological performance [226,262,263,264,265,266,267].

Among the various alloy systems investigated, Mg was one of the earliest and most extensively studied alloying elements for LPBF-processed Zn. Owing to its high solid-solution strengthening capability and the formation of intermetallic phases such as Mg_2_Zn_11_ and MgZn_2_, Mg additions substantially increased the strength of Zn alloys. Qin et al. [263] fabricated LPBF Zn–Mg alloys with relative densities above 99.5% and demonstrated that moderate Mg additions improved both hardness and strength through grain refinement and precipitation strengthening. However, increasing Mg content also promoted the formation of brittle intermetallic phases, resulting in a progressive reduction in ductility. Similar trends were reported by Voshage [262], highlighting the persistent challenge of balancing strength and plasticity in Zn–Mg systems. Recent work has further demonstrated that the mechanical behavior of LPBF Zn–Mg alloys is strongly governed by their unique additively manufactured microstructures.

To overcome the ductility limitations associated with binary Zn–Mg alloys, researchers subsequently explored more complex alloy systems. Shuai et al. [264] introduced Ag into Zn–Mg alloys fabricated by selective laser melting and reported the formation of unique spiral eutectic structures consisting of α-Zn and MgZn_2_ phases. These eutectic structures altered crack propagation paths and promoted energy dissipation during deformation, leading to simultaneous improvements in strength and ductility while maintaining good biocompatibility.

Silver has also been investigated as an independent alloying element in LPBF Zn alloys. Shuai et al. [264] reported that Ag additions promoted constitutional undercooling and stimulated heterogeneous nucleation during solidification, leading to significant grain refinement. At higher Ag contents, AgZn_3_ precipitates further contributed to strengthening through precipitation hardening. As a result, both compressive strength and hardness were substantially enhanced compared with pure Zn.

Another promising strategy involved Cu alloying. Lan et al. [265] fabricated LPBF Zn–2Cu alloys and demonstrated that the rapid solidification conditions of LPBF generated ultrafine equiaxed grains with an average size of only ~0.2 μm. The combined effects of grain refinement supersaturated solid solution strengthening, and precipitation of ε-CuZn_5_ and γ-Cu_5_Zn_8_ phases increased the yield and tensile strengths to approximately 145 MPa and 188 MPa, respectively. In addition to mechanical strengthening, Cu alloying offers potential antibacterial functionality, which is highly attractive for orthopedic applications.

More recently, multi-component alloy systems have been developed to achieve a more favorable balance between strength and ductility. Waqas et al. [266] reported a LPBF Zn–0.4Li–0.4Cu alloy exhibiting a tensile strength of 296 MPa and a yield strength of 260 MPa, representing one of the highest strength levels reported for additively manufactured biodegradable Zn alloys. The remarkable strengthening was attributed to the synergistic effects of grain refinement, solid-solution strengthening, and the precipitation of CuZn_4_ and ZnLi_4_ phases. Importantly, the alloy maintained a uniform degradation behavior and a relatively low corrosion rate, making it attractive for biodegradable stent applications. In parallel, Mn-containing alloys have attracted increasing attention because Mn can promote both grain refinement and biological functionality. Huang et al. [267] recently developed LPBF Zn-1Mn and Zn–1Mn–0.4Mg alloys with unique heterogeneous microstructures containing dispersed MnZn_13_ and Mg_2_Zn_11_ phases. The ternary Zn–Mn–Mg alloy achieved an unprecedented combination of strength and ductility among additively manufactured biodegradable Zn alloys, exhibiting a yield strength of 213.5 MPa, ultimate tensile strength of 289 MPa, and elongation exceeding 20%. Furthermore, the alloy demonstrated a moderate biodegradation rate (~0.15 mm/year) together with enhanced osteogenic activity, indicating that carefully designed multi-component alloy systems can simultaneously optimize mechanical, corrosion, and biological performance [267].

#### 5.3.4. Processing and Design of Porous Zn-Based Structures

Porous Zn-based structures are mainly developed for orthopedic and bone-regeneration applications, where controlled porosity is used to reduce stiffness, improve tissue ingrowth, enhance nutrient transport, and minimize stress shielding while preserving the moderate degradation behavior of Zn-based systems. Unlike dense Zn components, porous scaffolds are governed not only by alloy composition and microstructure but also by pore size, porosity, interconnectivity, strut thickness, surface condition, and scaffold topology. Therefore, this section focuses on the main fabrication routes for porous Zn-based materials, including powder metallurgy, replication methods, and additive manufacturing, with emphasis on how each route controls scaffold architecture, degradation behavior, and mechanical compatibility [19,20,55,56].

##### Powder Metallurgy

In powder metallurgy, alloy powders are mixed with space-holder particles, compacted, sintered, and subsequently processed to remove the space-holder phase [19,268,269]. Key processing parameters include the size and morphology of Zn powders, the type and size of space-holder particles (which determine pore size), compaction conditions (temperature, pressure, and holding time), sintering temperature and duration, total porosity, and pore interconnectivity [19,270]. The main advantages of this method are its relatively good control over pore size and porosity level, the ability to produce interconnected pore networks, and the formation of relatively large pores suitable for tissue ingrowth. However, improper process control may result in interparticle defects and incomplete densification [19,268,269]. For porous Zn scaffolds fabricated by powder metallurgy, porosity levels between 40 and 60% are generally considered optimal for balancing mechanical strength, permeability, and degradation behavior, while porosities of 40–50% are regarded as particularly promising for bone scaffold applications [19,269,271]. Most studies have focused on pure Zn and, to a lesser extent, Zn–Mg systems [19,270].

##### Replication Method

In the replication method, a polymer foam template is coated with a metallic slurry or suspension, after which the polymer is removed by thermal burnout, leaving behind a metallic skeleton that is subsequently sintered [272]. The most important parameters include the pore size of the initial foam, foam removal temperature, sintering conditions, slurry viscosity, and the number of coating cycles [272]. This method can produce highly interconnected porous structures that closely resemble the architecture of cancellous bone, making it particularly attractive for scaffold applications [19,268,272]. However, limitations include significant shrinkage during processing, limited control over precise pore geometry, potential cracking during foam burnout, and lower mechanical strength compared with LPBF-produced scaffolds [262,272].

##### Additive Manufacturing of Porous Zn-Based Scaffolds

In recent years, Selective Laser Melting (SLM) and Laser Powder Bed Fusion (LPBF) have become highly attractive technologies for manufacturing porous Zn-based scaffolds because they enable precise control of pore architecture, pore size, porosity gradients, and biomimetic structures [56,262,273]. The most important processing parameters include laser power, scanning speed, hatch spacing, and atmosphere/oxygen control [257,259,273]. Process conditions must provide sufficient energy for complete melting while avoiding excessive Zn evaporation, compositional changes, keyhole porosity, and poor surface quality. An energy density range of approximately 60–135 J/mm^3^ has been reported to achieve high relative densities (~99.5%) and acceptable forming quality, indicating a relatively narrow processing window [257,259,273]. Therefore, careful optimization of laser power, scanning speed, hatch spacing, layer thickness, and shielding gas flow is required to obtain high density, suitable microstructures, desirable mechanical properties, and controlled degradation behavior [256,257,273].

While dense Zn components are mainly developed for temporary fixation devices and load-bearing applications, porous Zn scaffolds are specifically designed for bone regeneration, where implant architecture becomes as important as the material itself. In these systems, pore size, porosity, interconnectivity, and topology directly influence mechanical compatibility, degradation kinetics, mass transport, and biological performance [19,56,258,263]. Consequently, the emergence of additive manufacturing has shifted scaffold development from simple material optimization toward the integrated design of both composition and architecture [56,263,274].

As illustrated in Figure 4, LPBF enables precise control over scaffold geometry, allowing the fabrication of periodic lattice structures, triply periodic minimal surface (TPMS) architectures, and functionally graded porous implants. This design freedom provides an additional degree of control that is not available in conventional processing routes, enabling the simultaneous tailoring of mechanical properties, degradation behavior, and biological response [55,56,258,274].

Initial studies on additively manufactured porous Zn scaffolds primarily focused on demonstrating manufacturing feasibility and achieving mechanical properties compatible with cancellous bone. These investigations showed that increasing porosity reduces elastic modulus and compressive strength while improving permeability and tissue accessibility, thereby reducing the risk of stress shielding. At the same time, the larger specific surface area of porous structures accelerates ion release and degradation relative to dense materials, highlighting the strong coupling between architecture and biodegradation behavior [19,56,258].

More recently, research has increasingly focused on the influence of scaffold topology. Shi et al. [55] systematically compared LPBF-fabricated Zn-Mn-Mg scaffolds with Diamond, FCC, Gyroid, and Schwarz-P architectures and demonstrated that topology alone can significantly influence both degradation behavior and mechanical performance. Beam-based structures such as Diamond and FCC exhibited relatively uniform degradation and higher permeability owing to their open-channel morphology. In contrast, TPMS architectures such as Gyroid and Schwarz-P generated more complex mass-transport pathways and promoted localized accumulation of degradation products, resulting in distinct degradation kinetics. These findings demonstrated that scaffold topology can serve as an effective design parameter for controlling biodegradation independently of alloy composition [55].

Architecture also plays a critical role in determining mechanical behavior. TPMS-based structures generally exhibit smoother stress distributions and higher energy-absorption capability than conventional lattice structures, whereas beam-based architectures often provide superior permeability and easier control of porosity. Consequently, scaffold optimization requires balancing stiffness, strength, permeability, and degradation rate according to the targeted clinical application [55,56]. Recent studies on LPBF-fabricated Zn-Mg-Sr porous scaffolds demonstrated that appropriately designed architectures can achieve compressive properties approaching those of trabecular bone while maintaining favorable degradation behavior and excellent cytocompatibility [274].

Beyond mechanical and corrosion considerations, porous Zn scaffolds provide significant biological advantages through their interconnected pore networks, which facilitate cell migration, nutrient transport, vascularization, and new tissue ingrowth. Furthermore, controlled Zn-ion release has been associated with enhanced osteogenesis, angiogenesis, and antibacterial activity [19,56,263]. Several in vitro and in vivo studies have shown that optimized scaffold architectures can significantly improve bone regeneration even when identical alloy compositions are employed, emphasizing the importance of structural design in determining biological performance [19,56,222,271].

Overall, the development of additively manufactured porous Zn scaffolds has evolved from the fabrication of simple porous structures toward architecture-driven design strategies [55,56,258]. Current research increasingly recognizes topology, porosity, and interconnectivity as engineering variables with an influence comparable to alloy composition itself. Future developments are therefore expected to combine alloy design, LPBF process optimization, topology engineering, functionally graded architectures, and patient-specific manufacturing to achieve multifunctional biodegradable implants with simultaneously optimized mechanical, degradation, and biological performance [55,56,263,274].

Compared with dense additively manufactured Zn components, porous Zn-based scaffolds should be evaluated primarily through topology-dependent degradation and mechanical-integrity retention rather than processing feasibility alone. Increasing porosity and pore interconnectivity can enhance tissue ingrowth, nutrient transport, and surface-area-driven degradation, but excessive porosity may reduce compressive strength and accelerate premature mechanical loss. In addition, additively manufactured defects, unmelted particles, rough strut surfaces, and microstructural heterogeneity can create local electrochemical differences and promote non-uniform corrosion. Therefore, future porous Zn scaffold design should integrate alloy composition, AM processing parameters, pore architecture, surface condition, and post-processing treatments to achieve a balanced combination of degradation rate, load-bearing capacity, and biological performance [19,20,55,56,202].

## 6. Conclusions and Future Perspectives

Biodegradable metals are promising alternatives to permanent implants because they can support tissue during healing and then gradually degrade, avoiding the need for removal surgery. Fe, Mg, and Zn-based alloys each have different strengths and limitations. Fe alloys have high strength but degrade too slowly. Mg alloys have a bone-like modulus and good biocompatibility but corrode too fast and produce hydrogen gas. Zn alloys show a more balanced degradation rate and do not generate significant hydrogen, but their strength, ductility, and fatigue resistance still need improvement. Recent progress in alloy design, thermomechanical processing, porous structures, and additive manufacturing has shown that the properties of biodegradable metals can be tailored by controlling composition, microstructure, secondary phases, and implant architecture. Additive manufacturing is especially promising because it enables patient-specific implants and porous scaffolds with controlled geometry and mechanical behavior. However, several challenges remain. Fe-based alloys require faster degradation without losing mechanical strength. Mg-based alloys require better control of rapid corrosion and hydrogen evolution. Zn-based alloys require further improvements in strength, ductility, fatigue resistance, and long-term degradation predictability. For all three systems, more attention is needed on long-term in vivo studies, corrosion–fatigue behavior, standardized testing methods, and the biological effects of degradation products.

The future development of biodegradable Fe-, Mg-, and Zn-based metals should focus on the combined control of processing, microstructure, mechanical properties, and degradation behavior. The evidence reviewed in this article indicates that processing route, microstructural evolution, secondary-phase distribution, crystallographic texture, scaffold architecture, and testing environment collectively determine degradation behavior and mechanical-integrity retention. Therefore, the next generation of biodegradable metallic implants should be designed using an integrated processing–structure–property–degradation framework. Therefore, thermomechanical processing and additive manufacturing should be used not only as fabrication routes, but also as tools to tailor degradation, mechanical stability, and biological response throughout the healing period [12,14,20,165,275].

## Figures and Tables

**Table 1:** Mechanical benchmark criteria for biodegradable orthopedic implants and vascular stents based on the literature and standards [8,9].

Parameter	Vascular Stents	Orthopedic Fixation Devices	Importance
Yield strength (YS)	>200 MPa	>230 MPa	Radial support/load-bearing capability
Ultimate tensile strength (UTS)	>300 MPa	>300 MPa	Structural integrity
Elongation to failure	15–20%	>15%	Deformation without fracture
Elastic recoil	<4%	—	Prevent vessel collapse/restenosis
Elastic modulus	High enough for radial support	Close to cortical bone (10–20 GPa)	Mechanical compatibility
Mechanical integrity retention	3–6 months	3–6 months	Maintain support during healing
Full degradation	1–2 years	1–2 years	Avoid secondary surgery

**Table 2:** Comparison of the typical mechanical properties of cortical bone and pure biodegradable metallic systems commonly investigated for biomedical applications [8,39].

Property	Cortical Bone	Pure Fe	Pure Mg	Pure Zn
Elastic modulus (GPa)	10–30	210	41–45	90–110
Yield strength (MPa)	100–200	120–180	20–65	20–50
Ultimate tensile strength (MPa)	130–200	200–250	90–150	30–120
Elongation (%)	5–20	30–50	3–10	0.5–5

**Table 3:** Representative biodegradable metallic systems approaching mechanical benchmark requirements and their remaining limitations.

Material System	Representative Alloy/Processing Route	Reported Advantage	Remaining Limitation	Ref.
Fe-based alloys	Fe–Mn, Fe–Mn–C, Fe–Mn–Si, Fe–Mn–Ag processed by rolling, drawing, or additive manufacturing	High strength, good ductility, and suitable load-bearing capacity	Very slow degradation, high elastic modulus, and possible long-term corrosion-product retention	[12,40,41]
Mg-based alloys	WE43, Mg–Zn–Ca, Mg–Zn–Zr, Mg–RE alloys processed by extrusion, rolling, or heat treatment	Bone-like modulus, improved strength after processing, and good biological potential	Rapid corrosion, hydrogen evolution, local alkalization, and premature mechanical loss	[2,13,14]
Zn-based alloys	Zn–Mg, Zn–Li, Zn–Mn, Zn–Cu, Zn–RE processed by extrusion, rolling, ECAP, or LPBF	Intermediate degradation rate, no hydrogen evolution, and improved strength/ductility after processing	Microstructure-dependent and sometimes heterogeneous corrosion; pure Zn is weak in the as-cast state	[8,9,10]

**Table 4:** Comparison of the electrochemical and corrosion characteristics of Fe, Mg, and Zn-based biodegradable metallic systems [6,42,43].

Parameter	Fe-Based Systems	Mg-Based Systems	Zn-Based Systems
Standard electrode potential (V vs. SHE)	−0.44	−2.37	−0.76
Relative corrosion rate	Very slow	Very fast	Moderate
Main corrosion products	Fe oxides/hydroxides	Mg(OH)_2_, MgO	ZnO, Zn(OH)_2_, Zn phosphates
Hydrogen gas evolution	Very limited	Significant	Negligible
Typical degradation mode	Passive oxide-controlled	Often localized/pitting	Microstructure-dependent

**Table 5:** Influence of common physiological test media on the corrosion behavior of biodegradable Mg-, Zn-, and Fe-based metals [8,12,44,45,46,47].

Test Medium	Main Chemical Feature	Mg-Based Metals	Zn-Based Metals	Fe-Based Metals	Main Limitation
SBF	Rich in inorganic ions; promotes apatite-like precipitation	May form Mg(OH)_2_ and calcium-phosphate layers; pH can increase rapidly	May form zinc phosphate, carbonate, or oxide/hydroxide products; degradation may appear more stable	Can promote phosphate/oxide-containing corrosion products but degradation remains slow	Does not fully reproduce proteins, cells, and dynamic in vivo fluid flow
HBSS/Hank’s solution	Contains chloride, calcium, phosphate, and bicarbonate depending on formulation	Chloride promotes Mg dissolution, while Ca/P species may form partially protective deposits	Can reveal microstructure-dependent corrosion and phosphate-containing products	Usually shows slow degradation due to oxide/hydroxide layer formation	Results depend strongly on buffering and whether the solution contains Ca^2+^/Mg^2+^
PBS	High phosphate-buffering capacity	May suppress or modify Mg corrosion by phosphate-containing deposits; not always representative for Mg	Can promote zinc phosphate formation and alter apparent corrosion rate	May enhance phosphate-rich film formation and apparent passivation	Strong buffering and high phosphate may underestimate or misrepresent degradation
DMEM	Contains salts, glucose, amino acids, vitamins, and often proteins/serum	Organic components and proteins can change film formation, pH evolution, and corrosion rate	More biologically relevant for cell-related degradation response	Proteins and organic molecules may affect Fe ion release and corrosion-product stability	Composition changes with serum addition and incubation conditions
Ringer’s solution	Simple chloride-rich salt solution	Can accelerate Mg corrosion because Cl^−^ attacks Mg(OH)_2_ layers	Useful for evaluating chloride-driven Zn corrosion	Fe corrosion remains relatively slow but chloride can destabilize surface films	Too simple; lacks phosphate, proteins, and organic components
Tyrode’s solution	Contains NaCl, KCl, CaCl_2_, MgCl_2_, bicarbonate/phosphate/glucose depending on formulation	Can better represent ionic physiological conditions; pH and carbonate species affect Mg corrosion	May produce mixed oxide, hydroxide, carbonate, or phosphate products	Can affect oxide/hydroxide film stability and ion release	Different formulations make comparison between studies difficult

**Table 6:** Representative Fe-based bioresorbable alloy systems, alloying strategies, main effects, and remaining limitations.

Alloy System	Main Alloying Strategy	Processing/Fabrication Route	Main Reported Effect	Remaining Limitation	Ref.
Fe–Mn	Austenite stabilization and magnetic response control	Casting, rolling, drawing, swaging, LPBF/SLM	Improved ductility, reduced ferromagnetism, good load-bearing potential	Degradation still slower than desired; possible retention of corrosion products	[51,58]
Fe–Mn–C	Solid-solution strengthening and TWIP/TRIP-assisted deformation	Hot/cold rolling, annealing, thermomechanical processing	Increased strength and work hardening; improved mechanical integrity	Carbide formation may promote micro-galvanic corrosion and reduce corrosion uniformity	[59,60,61,62,95]
Fe–Mn–Si	ε-martensite formation and shape-memory behavior	Rolling, annealing, solution treatment, quenching	Tunable phase transformation, reduced Young’s modulus, and shape-memory response	Complex phase control; corrosion response depends strongly on heat treatment and martensite fraction	[64,65,66,67,68,69,96,97,98]
Fe–Pd	Micro-galvanic degradation acceleration	Casting, thermomechanical processing	Nearly one-order increase in degradation rate while retaining high strength	High cost and possible concerns about noble-metal addition for large-scale implants	[70,71,72,73,99]
Fe–Ag/Fe–Cu	Cathodic second-phase formation and antibacterial functionality	Casting, powder metallurgy, rolling, AM-related routes	Accelerated corrosion through galvanic coupling; potential antibacterial activity	Localized corrosion risk depends on particle size, distribution, and ion release	[63,74,75,76,84,85,86,87]
Fe–Mn–Ca/Fe–Mn–Mg	Biofunctional alloying for bone-related applications	Casting, hot extrusion, thermomechanical processing	Potential osteogenic contribution and increased corrosion activity	Limited solubility in Fe; segregation and brittle secondary phases may promote localized degradation	[88,89,90,91,100]
Porous Fe/Fe–Mn	Architectural acceleration of degradation	Powder metallurgy, space-holder, sponge replica, LPBF/SLM, DIW	Increased surface area, lower stiffness, improved tissue ingrowth, and faster degradation than dense Fe	Reduced fatigue/load-bearing capacity at high porosity; degradation may still be insufficient	[16,53,101,102,103,104,105,106,107,108,109]
Fe–Mn–bioactive ceramic composites	Bioactive phase incorporation	DIW, extrusion-based 3D printing, sintering	Improved osteogenic response, biodegradability, and scaffold functionality	Processing complexity; need for stronger long-term in vivo validation	[24,110,111,112,113]

**Table 7:** Thermomechanical and manufacturing routes used for Fe-based biodegradable alloys and their main microstructural and corrosion-related effects.

Processing Route	Representative Alloy System	Processing Condition/Feature	Main Microstructural Effect	Effect on Properties/Corrosion	Remaining Limitation	Ref.
Hot rolling	Fe–Mn, Fe–Mn–Si, Fe–Mn–C, Fe–Mn–C–Ag	Typically applied at elevated temperatures, about 600–1100 °C depending on alloy system	Grain refinement, improved homogeneity, modification of γ-austenite/ε-martensite balance	Improves strength and ductility; may modify corrosion behavior through phase redistribution	Corrosion response remains strongly dependent on phase fraction and alloy chemistry	[63,64,65,97,114]
Cold rolling	Fe–Mn, Fe–Mn–C, Fe–Mn–Si	Plastic deformation at room temperature, often followed by annealing	Increased dislocation density, work hardening, texture development	Increases strength but may reduce ductility; corrosion behavior depends on strain level and annealing	Excessive deformation may reduce plasticity and promote localized corrosion	[57,59,63,64]
Rolling + annealing/solution treatment	Fe–Mn–Si, Fe–Mn–C, Fe–Mn–Si-based alloys	Rolling followed by recrystallization, solution treatment, quenching, or annealing	Controls grain size, residual stress, γ ↔ ε transformation, and carbide/second-phase evolution	Can tune Young’s modulus, shape-memory response, and electrochemical behavior	Requires precise control of temperature and phase transformation	[65,66,96,98,114]
Forging/hot compression/swaging	Fe–Mn, Fe–Mn–Si, Fe–Mn–C-based alloys	Bulk compressive deformation	Generates deformation bands, twins, refined grains, and phase changes	Improves homogeneity and mechanical strength; may affect degradation through defect density and phase redistribution	Limited systematic corrosion data compared with rolling and drawing	[51,63,67,114]
Wire and tube drawing	Fe–Mn-based stents and neurovascular devices	Severe shape reduction to produce thin wires or tubes	Produces fine geometries, high strength, and deformation texture	Important for radial strength and stent geometry	Intermediate annealing is often required to avoid excessive loss of ductility	[41,58,62,95]
ECAP/HPT	Fe–Mn–C and Fe–Mn–Si alloys	Severe plastic deformation	Produces ultrafine or nanostructured grains and high defect density	Strong strengthening and possible corrosion modification	Corrosion does not always improve because phase transformations may reduce micro-galvanic activity	[67,68,99]
Hot extrusion	Fe–Mn–Ca-based alloys	Less explored route; extrusion parameters still limited	Can improve densification, phase distribution, and microstructural alignment	Potentially useful for producing dense biodegradable Fe components	Systematic studies on extrusion temperature, ratio, and speed are still lacking	[88]

**Table 8:** Alloy-family-dependent effects of heat treatment on the microstructure and corrosion behavior of biodegradable Mg alloys.

Alloy Family	Typical Heat Treatment	Main Microstructural Change	Effect on Corrosion Behavior	Key Limitation	References
Mg–Al alloys	T4, T6, solution treatment, aging	Dissolution, redistribution, or precipitation of β-Mg17Al12	Discontinuous β-Mg17Al12 may act as a barrier, but continuous/cathodic β networks can promote galvanic corrosion	Response depends strongly on β-phase morphology and continuity	[165]
Mg–Zn/Mg–Zn–Zr alloys	T4, T6, extrusion + aging	Dissolution of Zn-rich phases during T4; precipitation during aging	T4 may improve corrosion resistance by homogenizing solute distribution; T6 may improve strength but can increase localized corrosion if precipitates are coarse or continuous	Balance between strengthening and corrosion resistance is difficult	[164,165]
Mg–Y–Nd–Zr/WE43-type alloys	Solution treatment, aging, T6	Modification of RE-rich β′, β1, and grain-boundary precipitates	Proper aging may improve strength and film stability, but non-uniform RE-rich precipitates can promote localized attack and delayed hydrogen evolution	In vivo corrosion may differ from short-term in vitro tests	[166,167]
Mg–Gd-based alloys	Solution treatment and aging	Formation and evolution of Gd-rich precipitates	Can improve mechanical strength and sometimes corrosion resistance by modifying precipitate distribution	Excessive precipitates may intensify micro-galvanic corrosion	[165]
Mg–Nd-based alloys	T4, T6, aging	Redistribution of Nd-rich intermetallics and precipitates	Corrosion response depends on precipitate size, distribution, and matrix/second-phase potential difference	Limited systematic comparison among heat-treatment states	[165]
Mg–Ag/Mg–RE systems	Aging and precipitation hardening	Formation of strengthening precipitates and modified surface-film chemistry	May improve mechanical performance, but corrosion behavior remains highly dependent on precipitate chemistry and distribution	Biocompatibility and ion-release effects require careful evaluation	[165]

**Table 9:** Fabrication routes for porous Mg-based biodegradable scaffolds and their main architecture-related effects.

Fabrication Route	Main Feature	Advantage	Main Limitation	References
Powder metallurgy/space-holder	Temporary particles such as NaCl, urea, or ammonium bicarbonate are used to create pores after compaction and sintering	Relatively simple control of pore size, porosity, and interconnectivity	Powder oxidation, incomplete sintering, residual MgO, and non-uniform local corrosion	[17,18,186]
LPBF/SLM	Layer-by-layer laser melting enables ordered porous structures and controlled lattice geometries	High architectural precision, patient-specific design, and controlled strut/pore geometry	Mg evaporation, oxidation, flammability, spattering, and narrow processing window	[185,191,192]
Solvent-cast 3D printing	Mg-based scaffolds are produced through printed polymer/particle mixtures followed by post-processing	Allows complex porous geometries without direct laser melting of Mg powder	Binder removal, shrinkage, residual contamination, and limited densification control	[193]
Infiltration casting	Molten Mg infiltrates a sacrificial porous template	Good metallurgical continuity and interconnected open-cell structures	Difficult control of melt infiltration, oxidation, template removal, and residual contamination	[197,198]
Sacrificial template/replica methods	Polymer or salt templates define the final pore network	High interconnectivity and cancellous-bone-like architecture	Shrinkage, cracking during template removal, and limited precision of pore geometry	[196,199]
Surface-coated porous Mg	Porous Mg is combined with HA, calcium-phosphate, fluoride, oxide, or PEO coatings	Reduces initial corrosion rate and improves bioactivity	Long-term protection depends on coating adhesion, degradation stability, and coating/substrate interaction	[27,185,192]

## Data Availability

No new data were created or analyzed in this study. Data sharing is not applicable to this article.
